# Chlorotoxin targets ERα/VASP signaling pathway to combat breast cancer

**DOI:** 10.1002/cam4.2019

**Published:** 2019-02-25

**Authors:** Ying Wang, Kai Li, Song Han, Yi‐hao Tian, Peng‐chao Hu, Xiao‐long Xu, Yan‐qi He, Wen‐ting Pan, Yang Gao, Zun Zhang, Jing‐wei Zhang, Lei Wei

**Affiliations:** ^1^ Department of Pathology and Pathophysiology, Hubei Provincial Key Laboratory of Developmentally Originated Disease, School of Basic Medical Sciences Wuhan University Wuhan Hubei China; ^2^ Department of Oncology Xiangyang No.1 People’s Hospital, Hubei University of Medicine Xiangyang Hubei China; ^3^ Department of Anatomy, School of Basic Medical Sciences Wuhan University Wuhan Hubei China; ^4^ Department of Breast and Thyroid Surgery Zhongnan Hospital Hubei Key Laboratory of Tumor Biological Behaviors, Hubei Cancer Clinical Study Center Wuhan University Wuhan Hubei China

**Keywords:** breast cancer, chlorotoxin, ERα, treatment, VASP

## Abstract

Breast cancer is one of the most common malignant tumors among women worldwide. About 70‐75% of primary breast cancers belong to estrogen receptor (ER)‐positive breast cancer. In the development of ER‐positive breast cancer, abnormal activation of the ERα pathway plays an important role and is also a key point leading to the failure of clinical endocrine therapy. In this study, we found that the small molecule peptide chlorotoxin (CTX) can significantly inhibit the proliferation, migration and invasion of breast cancer cells. In in vitro study, CTX inhibits the expression of ERα in breast cancer cells. Further studies showed that CTX can directly bind to ERα and change the protein secondary structure of its LBD domain, thereby inhibiting the ERα signaling pathway. In addition, we also found that vasodilator stimulated phosphoprotein (VASP) is a target gene of ERα signaling pathway, and CTX can inhibit breast cancer cell proliferation, migration, and invasion through ERα/VASP signaling pathway. In in vivo study, CTX significantly inhibits growth of ER overexpressing breast tumor and, more importantly, based on the mechanism of CTX interacting with ERα, we found that CTX can target ER overexpressing breast tumors in vivo. Our study reveals a new mechanism of CTX anti‐ER‐positive breast cancer, which also provides an important reference for the study of CTX anti‐ER‐related tumors.

## 1 INTRODUCTION

Breast cancer is one of the most common malignant tumors among women worldwide. Currently, more than 400 000 patients die of breast cancer every year, and the incidence rate is increasing year by year.^1^. About 90% of breast cancer patients die from tumor metastasis.^2^ In the development of breast cancer, the estrogen receptor (ER)‐mediated signaling pathway is an important factor regulating the proliferation and migration of breast cancer cells.^3^ Therefore, exploring the mechanism of the ER signaling pathway and exploring drugs that target the ER signaling pathway are important strategies for the treatment of breast cancer.

ER is a functional receptor that mediates the action of estrogens.^4^ Estrogen is involved in the regulation of various physiological functions in the reproductive and nonreproductive systems through ER, and is closely related to various clinical diseases, especially breast cancer.^5^ According to the expression of ERs, breast cancer can be divided into two types: ER positive and ER negative. According to statistics, about 70‐75% of primary breast cancers belong to ER‐positive breast cancer.^6^ ER mainly has two different subtypes, including α and β, which are encoded by two independent genes.^7^ ERα receptors are mainly distributed in the uterus, breast, placenta, liver, central nervous system, cardiovascular system, bone tissue. ERα is a key receptor for estrogen in normal breast.^8^ Current studies have shown that ERα activation works through intracellular signaling molecules such as mitogen‐activated protein kinase and phosphoinosmde‐3‐kinase, which plays an important role in regulating cell proliferation^9^; The ERα in the nucleus mainly plays as a transcription factor, thereby regulating the activation of downstream target genes.^10^ Some studies have also shown that ERα not only regulates cell proliferation, but also regulates tumor cell migration and invasion.^11^ Therefore, endocrine therapy targeting ERα is one of the conventional treatment measures for hormone‐sensitive breast cancer, but with the prolongation of treatment progress, some patients' sensitivity to endocrine therapy will gradually decrease or even resist.^12^

Small molecule peptides are becoming a hot research topic in the medical and pharmaceutical fields. Among them, the novel antitumor active polypeptide has the characteristics of high affinity, strong specificity, and low adverse reaction, and most of them also have the property of selectively targeting tumor cells, and thus have very important development value in clinical application.^13^, ^14^ Chlorotoxin (CTX) is a short peptide of scorpion toxin isolated from the venom of the Israel golden carp, consisting of 36 amino acids.^15^ Studies have shown that CTX can bind to proteins on the surface of tumor cells, allowing them to specifically bind to tumor tissues. For example, CTX can preferentially bind to glioma cells which express MMP2 protein rather than normal cells.^16^, ^17^ CTX can not only bind glioma cells, but also a variety of tumor cells such as lung cancer cells, prostate cancer cells and melanoma.^18^, ^19^ CTX has also been shown to have potent antiangiogenic activity^20^ and can inhibit migration and invasion of a variety of tumor cells.^21^, ^22^ Because of these biological characteristics, CTX is constantly being developed as a new diagnostic tool or adjuvant therapy, and has a broad application space in clinical medicine.^23^ Another study reported that CTX has different degrees of inhibitory effect on the proliferation and migration of common female tumor cells such as cervical cancer, endometrial cancer, and ovarian cancer.^24^ However, there are few reports on CTX inhibition of breast cancer cells, and the mechanism is not clear.

In the present study, we found that CTX can significantly inhibit the proliferation, migration, and invasion of breast cancer cells, and its mechanism may be related to the ability of CTX to target ERα/VASP signaling pathway. More importantly, in in vivo study, we found that CTX is more likely to target breast cancer cells overexpressing ER, and these findings will provide new strategies for the treatment of breast cancer.

## 2 MATERIAL AND METHODS

### 2.1 Plasmids construction

The full‐length coding sequence of CTX polypeptide (P45639, NCBI) was from NCBI database. Considering the rare codon contained in the coding sequence, the nucleotide coding sequence after reverse translation optimization is: ATGTGTATGCCGTGCTTCACTACCGATCACCAGATGGCACGTAAATGTGACGATTGCTGTGGTGGCAAAGGTCGTGGTAAATGCTACGGTCCGCAGTGTCTGTGCCGTTGA.The primers of CTX gene were designed to carry out overlapping PCR. The overlapping primers were CTX‐F1: CGTCGTTGTGCCGATTGCTGTGGTGGCAAAGGTCGTGGTAAATGCTACGG; CTX‐F2: GCCGGATCCCCGATGACGATGACAAGATGTGTATGCCGTGCTTCACTACC; CTX‐R1: CAATCGTCACATTTACGTGCCATCTGGTGATCGGTAGTGAAGCACGGCAT; CTX‐R2: GCCCTCGAGTCAACGGCACAGACACTGCGGACCGTAGCATTTACCACGAC.

The primers of pET‐28a‐ERα‐LBD were ERα‐LBD‐F: CCCTCGAGCTGGCCTTGTCCCTGACGGCCGACC; ERα‐ LBD‐R: CGCCATATGCAGCTAGTGGGCGCATGTAGGCGGT. The conjugated product was transferred into DH5α‐sensitive bacteria. A recombinant plasmid Miniprep Kit (Axygen, New York, NY, USA) was used to extract the recombinant plasmid. The vector was identified by double enzyme digestion (BamH I, Xho I) and sequencing. The successful vector was named pGEX‐6p‐1‐CTX.

### 2.2 Affinity chromatography

Affinity chromatography can be used to isolate and purify GST‐CTX fusion protein. First, pGEX‐6p‐1‐CTX was transformed into Transetta competent cells. isopropyl β‐D‐thiogalactoside (IPTG) (1 mmol/L; Sigma, St. Louis, MO, USA) was used to induce the expression of GST‐CTX fusion protein. GST‐CTX fusion protein was purified by affinity chromatography (0.22‐μm microporous filter membrane at a rate of 0.5 mL/min, then washed by PBS at a rate of 1 mL/min and then purified by GSH eluate GST‐CTX fusion protein at 1.5 mL/min). The GST‐CTX protein eluate was detected by Tris‐Tricine‐SDS‐PAGE electrophoresis, and then concentrated using Millipore Amicon‐Ultra‐15 (MWCO10kD) ultrafiltration centrifuge tubes.

### 2.3 Reversed phase high‐performance liquid chromatography

GST‐CTX protein was digested by EK at 4°C, 12 000 rpm for 5 minutes. The collected CTX solution was separated and purified by reversed‐phase high‐performance liquid chromatography (RP‐HPLC) (Agilent Technologies, USA). The condition was as follows: the stationary phase was C18 reversed‐phase column (SinoChrom ODS‐AP 300 μm 10.0 × 250 mm). The mobile phase consists of solution C (0.1 TFA) and solution D (0.1% TFA). Then, 90% acetonitrile was added. The sample amount is 1 mL. The elution gradient is 60 minutes linear gradient, the initial solution C, solution D is 95% and 5%, and at the end, solution C and solution D are 5% and 95%, respectively. The flow rate is 5 mL/min and the wavelength is 230 nm. In addition, the molecular weight of the purified CTX protein is detected by MALDI‐TOF‐MS At last, the RP‐HPLC collected liquid was lyophilized, and then dissolved in 500 μL of precooled sterile ultrapure water, and dispensed by 100 mg/tube, stored at −80°C.

### 2.4 Cell culture

The cell lines used for this study are including the breast cancer cell MCF‐7, MDA‐MB‐231, and T47D. The cell lines were purchased from the China Center for Type Culture Collection (CCTCC, Chinese Academy of Sciences, Shanghai, China). MCF‐7 and MDA‐MB‐231 were cultured in DMEM medium (HyClone, Waltham, MA, USA) supplemented with 10% fetal bovine serum (FBS; Gibco, Milano, Italy), 100 U/mL penicillin, and 100 mg/mL streptomycin. T47D cells were cultured in RPMI‐1640 medium (HyClone, USA) supplemented with 10% FBS (Gibco), 100 U/mL penicillin, and 100 mg/mL streptomycin.

### 2.5 CCK‐8 assay

A CCK‐8 assay was used to determine cell proliferation. For the CCK‐8 assay (Cell counting kit‐8, Dojindo Laboratories, Shanghai, China), cells (3 × 10^3^ cells/well) in the logarithmic growth phase were cultured in 96‐well plates and incubated for 0 to 48 hours. Then, cells were treated with the concentration gradient and time gradient treatment of CTX. After the treatment period, 10 µL of CCK‐8 was added and continued to culture 2 hours. The value of OD450 was measured by an automated microplated reader (BioTek, Winooski, VT).

### 2.6 Wound healing assay

Cells (2 × 10^5^ cells/mL) were seeded in a six‐well plate and cultured. Until the cells achieved 80‐90% confluence, cells were scratched. Then, the cells were washed twice with phosphate‐buffered saline (PBS). After continued to culture 48 hours in the serum‐free medium containing different concentrations of CTX, cells were fixed and photographed under a microscope (Olympus, Tokyo, Japan), the number of cells migrated into the scratched area was calculated.

### 2.7 Transwell assay

The experiment used some 24‐well plates and a polyvinyl‐pyrrolidone‐free polycarbonate filter (8‐µm pore size). Cells, at a density of 1 × 10^5^ in 100 μL of serum‐free medium, treated with different concentrations of CTX were added to the upper well, and 700 μL of culture medium with 20% FBS was added to the lower chamber. After incubation 20 hours, cells were fixed, stained, and counted the cell number in the surface of the lower chamber under light microscope (Olympus). The assay could be used to detect the ability cell invasion and migrate. Differently, when it was used to detect the ability of cell invasion, the chamber was coated with Matrigel Matrix (BD, Franklin Lakes, NJ, USA).

### 2.8 Orthotopic transplantation tumor nude mouse models

Orthotopic transplantation tumor nude mouse models were used to detect the effect of CTX on breast cancer in vivo. Cells (1 × 10^8^ cells/mL) were injected subcutaneously into the second breast pad of 4‐week‐old female Balb/c nude mice (purchased from Animal Research Center of Wuhan University/ABSL‐III Laboratory) to establish a nude mouse orthotopic transplantation tumor model. For the MCF‐7 tumor model, mice received subcutaneous implants of 60‐day slow release 0.72 mg of 17β‐estradiol pellets (Innovative Research of America) 1 day before receiving tumor cell inoculation. On the 20th day of tumor formation, CTX:Cy5.5 (Lumiprobe, Baltimore, MD, USA) was injected into the mice of test group by tail vein injection, and the control group was injected with the same dose of normal saline, this operation was done every 2 days. Then, the weight of nude mice and the volume of the tumor were observed and recorded; meanwhile, the distribution of CTX: Cy5.5 in tumor‐bearing mice was detected by the animal in vivo imaging system. Data statistics was continued until the tumor‐bearing mice were executed. Our study was approved by the Ethics Board of School of Basic Medical Sciences, Wuhan University.

### 2.9 H&E staining

Hematoxylin and eosin (H&E) staining can be used to observe the general morphological characteristics of various tissue or cell components. The fresh tumor tissue of nude mice was fixed with 4% formaldehyde. After dehydration of ethanol from low to high and transparent treatment, the tissue was embedded in paraffin, then cut into thin pieces and dried on the slide glass. After dewaxing with xylene and ethanol, it was dyed with hematoxylin for 5 minutes, then washed repeatedly after acid treatment, and then dyed with 1% eosin for 2 minutes. After dehydration and transparent treatment, it is sealed with neutral resin. The pathological changes of tissues can be observed under a microscope.

### 2.10 Reverse transcription and quantitative polymerase chain reaction

The mRNA expression level of gene was examined by reverse transcription and quantitative polymerase chain reaction (RT‐qPCR) (SYBR Green Supermix, Bio‐Rad, Shanghai, China) normalized to the expression of GAPDH. First, total RNA was extracted from cells using Trizol reagent (Applied Biosystem Inc, Foster City, CA) according to the manufacturer's protocol. The cDNA was obtained by a RevertAid^TM^ First Strand cDNA Synthesis Kit (Fermentas, Ontario, Canada). Then, the expression level of target genes was analyzed by qPCR. 5 µL SYBR Green PCR Master Mix(2×) , 2 µL forward and reverse primers (ERα forward: AATTCTGACAATCGACGCCAG, reverse: GTGCTTCAACATTCTCCCTCCTC; VASP forward: CTGG GAGAAGAACAGCACAACC, reverse: AGGTCCGAGTA TCACTGGAGC; GAPDH forward: CCCAGCCATCAG TATTCAG, reverse: GAGTTGGCACCGTTACAGTG.), appropriate amount of cDNA, and ddH2O were mixed to total volume of 10 µL. The conditions of qPCR consisted of the following: 95˚C for 3 minutes for denaturation; 95°C for 20 seconds for annealing; and 72°C for 5 minutes for extension, for 40 cycles. The relative expression of eachgene was calculated by the 2^‐∆∆Ct^ method. Each experiment was repeated three times.

### 2.11 Western blot

The protein was extracted with RIPA lysate, and quantified with a BCA Protein Assay Kit (Beyotime Biotechnology Co, Shanghai, China). The protein was bind to the polyvinylidene fluoride (PVDF) membrane by electrophoretic and transmembrane. After blocking the nonspecific sites on the membrane with 5% sealant, a primary antibody (including ERα dilution of 1:1000, VASP dilution of 1:1000, MMP2 dilution of 1:1000, GAPDH dilution of 1:5000) was used to incubate with the PVDF membrane overnight at 4°C. The membrane was incubated with the corresponding secondary antibody (dilution of 1:1000) for 1 hour at room temperature, and finally detected by ECL reagents (Tanon, Shanghai, China). The optical density of bands was measured by a computer‐assisted imaging analysis system (Tanon) and the relative protein expression levels were normalized to GAPDH.

### 2.12 Immunofluorescence

Immunofluorescence can be used to detect the intracellular distribution and subcellular localization of proteins. The sterilized circular glass slides were placed in a 24‐well plate and cultured at 37°C with the cells of logarithmic growth period seeded on the glass plates. When the cells density is about 60‐75%, cells were treated with CTX and cultured for 48 hours; meanwhile, a control group was set. After being fixed, transparent, and blocking treatment, the antibody (VASP dilution of 1:500, actin dilution of 1:500) was added and incubated overnight at 4°C. The next day, after washing the glass, the second antibody with fluorescent labeling (Dylight 594 AffiniPure Goat Anti‐Mouse IgG, Wuhan, China) was added for 2 hours, then washing and sealing the slide, and observing the staining results under fluorescence microscope (Olympus).

### 2.13 Pull‐down assay

The pull‐down assay can be used to detect the binding between proteins. Target protein‐GST fusion protein was affinity‐cured on glutathione affinity resin, and acted as a bait protein. When the cellular protein lysate goes through the column (Transgen, Beijing, China), the protein that interacts with target protein could be captured. Then, the conjugate is eluted and analyzed by SDS‐PAGE electrophoresis to confirm the interaction or screening between the two proteins. In this experiment, the pull‐down assay was detected the combination of CTX and ERα.

### 2.14 Fluorescence spectrum

There are many chromogenic amino acids in CTX. The binding sites and binding strength between CTX and ERα‐LBD can be studied by fluorescence quenching method. The endogenous fluorescence of ERα‐LBD was detected by a LS‐55 Luminescence spectrometer (Perkin‐Elmer Life Science, Fremont, CA, USA). The experimental conditions were as follows: excitation wavelength was 295 nm, slit of excitation and emission was 10 nm, scanning speed was 1000 nm/min, temperature was 25°C, and ERα‐LBD (0.2 μm) sample was 600 μL, microaddition of CTX solution.

### 2.15 Chromatin immunoprecipitation

Chromatin immunoprecipitation (ChIP) can be used to detect protein–DNA interactions. Adding 37% formaldehyde to the cells makes DNA and protein cross‐linked. DNA was fragmented by ultrasound, and protein and DNA cross‐links were coprecipitated by protein A/G and ERα antibody; IgG was used as a negative control. Then, SDS‐NaCl‐DTT buffer and proteinase K were added to reverse cross‐link. After purification of DNA by a PCR cleanup kit, the concentration was determined and PCR was used to identify the target DNA fragment. The primers of ChIP were ERα‐VASPpro‐ChIP‐F: TTTAGTCTACCCATTCTCCCA; ERα‐VASPpro‐ChIP‐R: GTCCTGACCTCCTTTACCTG.

### 2.16 Luciferase assay

Luciferase assay was used to detect the activity of gene promoter. In this experiment, plasmids (pGL3‐Basic, pGL3‐VASP2.1K, pEGFP‐ERα, and pGL3‐VASP2.1K‐mut) were constructed, and transfected into cells using transfection reagent (Invitrogen, Carlsbad, CA); pRL‐TK (Promega, Madison, WI) was as reference. At 48 hours after transfection, the relative luciferase activities were measured by a dual‐luciferase reporter assay kit (Promega) according to the manufacturer's guide. The primers of ERα overexpression plasmid was ERα‐pEGFP‐C1‐F: CGGAATTCCATGACCATGACCCTCCACACCAAAG; ERα‐pEGFP‐C1‐R: CGGGATCCTCAGACCGTGGCAGGGAAACCCTCT. The primers of pGL3‐VASP2.1K‐mut were ERα‐VASPpro‐mut‐F: AGGAACAGGGTTCA**TCCAG**TCCCAGAGTGGGT; ERα‐VASPpro‐mut‐R: ACCCACTCTGGGA**CTGGA**TGAACCCTGTTCCT.

### 2.17 Statistical analysis

Statistical analysis was performed using software SPSS. Each set of experiment was repeated three times. The data were expressed as the mean ± standard deviation. The variance analysis between groups was performed using a one‐way ANOVA. A value of *P < 0.05* was considered statistically significant.

## 3 RESULTS

### 3.1 Preparation and purification of CTX

**Figure 1:**
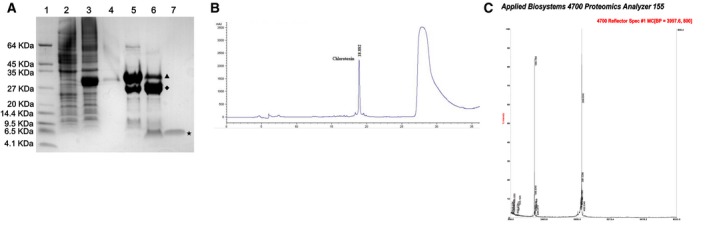
Preparation and purification of chlorotoxin. (A) The CTX recombination protein was isolated and identified by Tris‐Tricine‐SDS‐PAGE electrophoresis. Lane 1: Low Range Protein ladder marker, Lane 2: pGEX‐6p‐1‐CTX‐induced whole‐cell protein without IPTG, Lane 3: whole‐cell protein after induction of IPTG by pGEX‐6p‐1‐CTX, Lane 4: wash with PBS heteroprotein, Lane 5: concentrated GST‐CTX fusion protein after ultrafiltration, Lane 6: GST protein and CTX protein after digestion, Lane 7: HPLC purification of the obtained CTX protein. (B) The isolated CTX protein was purified by RP‐HPLC. (C) The molecular weight of the purified protein was determined by the MALDI‐TOF‐MS method. CTX, chlorotoxin; RP‐HPLC, reversed‐phase high‐performance liquid chromatography

In order to obtain the CTX protein, the prokaryotic expression vector pGEX‐6p‐1‐CTX containing the correct sequence was transferred into Transetta expressing bacteria, IPTG was used to induce the expression of GST‐CTX fusion protein, and the obtained product was detected by Tris‐Tricine‐SDS‐PAGE electrophoresis. The CTX protein was isolated and purified by RP‐HPLC, and then was identified by Tris‐Tricine‐SDS‐PAGE electrophoresis (Figure 1A,B), and finally, the CTX protein with purity above 95% was obtained. A small amount of the product purified from RP‐HPLC was taken, and the molecular weight of the protein was determined by MALDI‐TOF‐MS method, and the molecular weight was determined to be 3997.60 Da (Figure 1C), which was consistent with the calculated molecular weight. The results showed that the CTX protein was successfully purified.

### 3.2 CTX can inhibit the proliferation, migration, and invasion of breast cancer cells

**Figure 2:**
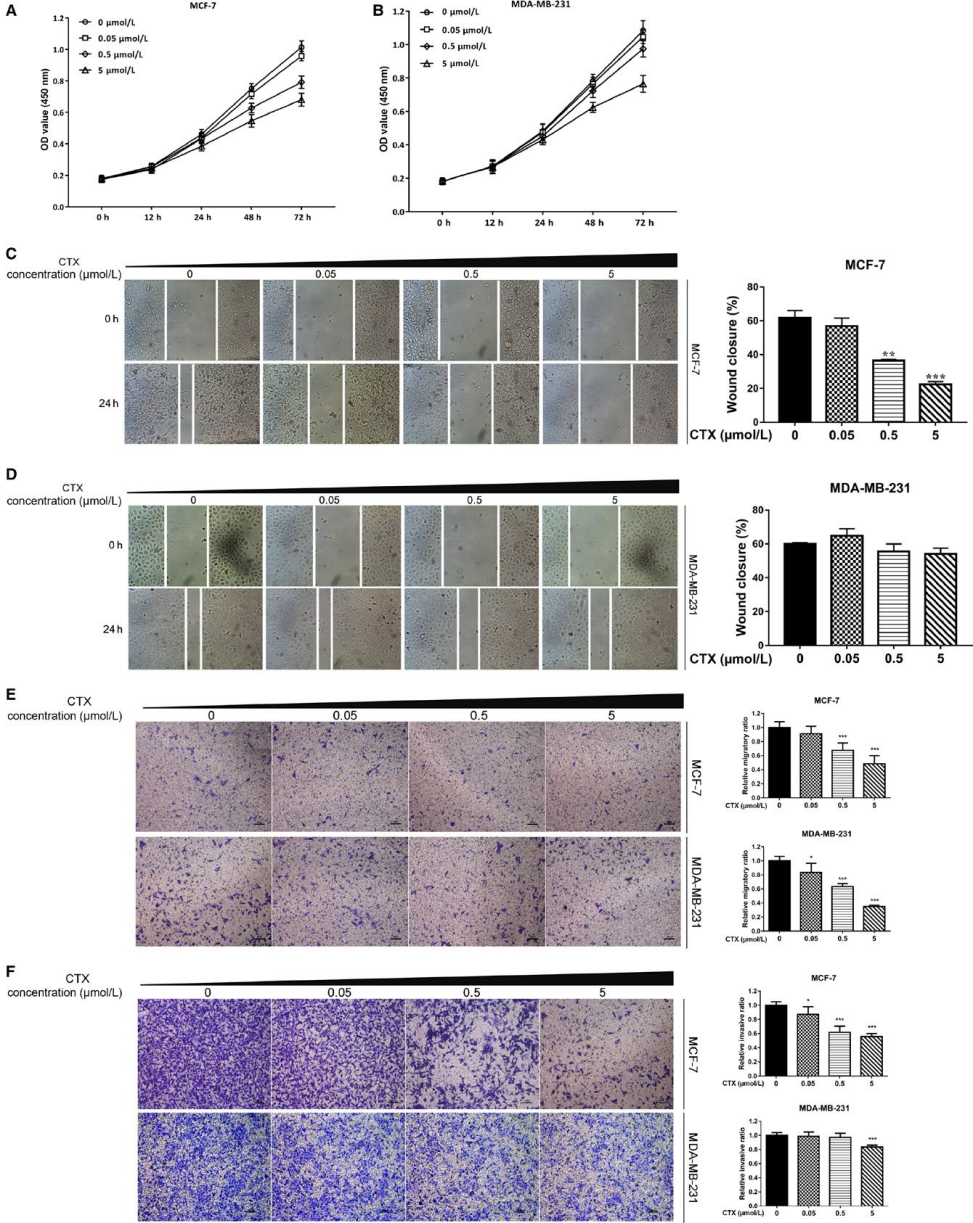
Chlorotoxin can inhibit the proliferation, migration, and invasion of breast cancer cells in a concentration‐ and time‐dependent manner. MCF‐7 (A) and MDA‐MB‐231 (B) cells were treated with CTX at 0, 0.05, 0.5, and 5 μmol/L for 0, 12, 24, 48, and 72 hours, respectively. Cell proliferation was measured by a CCK‐8 assay. MCF‐7 (C) and MDA‐MB‐231 (D) breast cancer cells were treated with 0, 0.05, 0.5, and 5 μmol/L CTX for 24 hours. Cell migration was tested by a wound healing assay. (E) MCF‐7 and MDA‐MB‐231 breast cancer cells were treated with 0, 0.05, 0.5, and 5 μmol/L CTX for 24 hours. Cell migration was tested by a transwell assay. (F) MCF‐7 and MDA‐MB‐231 breast cancer cells were treated with 0, 0.05, 0.5, and 5 μmol/L CTX for 24 hours. Cell invasion was tested by a transwell assay. *P < 0.05, **P < 0.01, ***P < 0.001. CTX, chlorotoxin

In order to observe the effect of different concentrations of CTX on the proliferation of breast cancer cells, MCF‐7 and MDA‐MB‐231 cells were treated with CTX at 0, 0.05, 0.5, and 5 μmol/L for 0, 12, 24, 48, and 72 hours, respectively. Cell proliferation was measured by the CCK‐8 assay. The results showed that in MCF‐7 cells and MDA‐MB‐231 cells, compared with the control group, when the cells were treated with 0.05, 0.5, and 5 μmol/L CTX, the cell proliferation was inhibited in a concentration‐ and time‐dependent manner (*P* < 0.05) (Figure 2A,B). Furthermore, another ER‐positive breast cancer cells T47D was also treated with 0, 0.05, 0.5, and 5 μmol/L CTX for 0, 12, 24, 48, and 72 hours, respectively. The results showed that the proliferation of T47D cells was also inhibited in a concentration‐ and time‐dependent manner (*P* < 0.05) (Figure S1A).

To observe the effects of different concentrations of CTX on the migration and invasion of breast cancer cell, MCF‐7 and MDA‐MB‐231 breast cancer cells were treated with 0, 0.05, 0.5, and 5 μmol/L CTX for 24 hours. Cell migration and invasion were tested by a wound healing assay and transwell assay. The results of the wound healing assay showed that in MCF‐7 and MDA‐MB‐231 cells, when treated with 0.05, 0.5, and 5 μmol/L CTX, the capacity of cell migration was decreased, and the inhibition rates were 9.9%, 43.6%, 65.8%, and 0%, 7.7%, 11.2%, respectively (*P* < 0.05) (Figure 2C,D). A transwell migration assay showed that in MCF‐7 and MDA‐MB‐231 cells, when treated with 0.05, 0.5, and 5 μmol/L CTX, the inhibition rates of cell migration were 12.7%, 38.2%, 44.3%, and 1.3%, 3.1%, 16.4%, respectively (*P* < 0.05) (Figure 2E). The results of transwell invasion assay showed that in MCF‐7 cells and MDA‐MB‐231 cells, when treated with 0.05, 0.5, and 5 μmol/L CTX, the invasive ability decreased, and the inhibition rates were about 8.9%, 32.6%, 51.7%, and 16.6%, 36.7%, 65.1%, respectively (*P* < 0.05) (Figure 2F). Furthermore, another ER‐positive breast cancer cells T47D was also treated with 0, 0.05, 0.5, and 5 μmol/L CTX for 36 hours. The migration and invasion of T47D cells were detected by transwell assay (Figure S1B). The results showed that CTX can inhibit the migration and invasion of MCF‐7, MDA‐MB‐231 and T47D breast cancer cells in a concentration‐dependent manner.

### 3.3 CTX can significantly inhibit the expression levels of ERα and VASP in breast cancer cells

**Figure 3:**
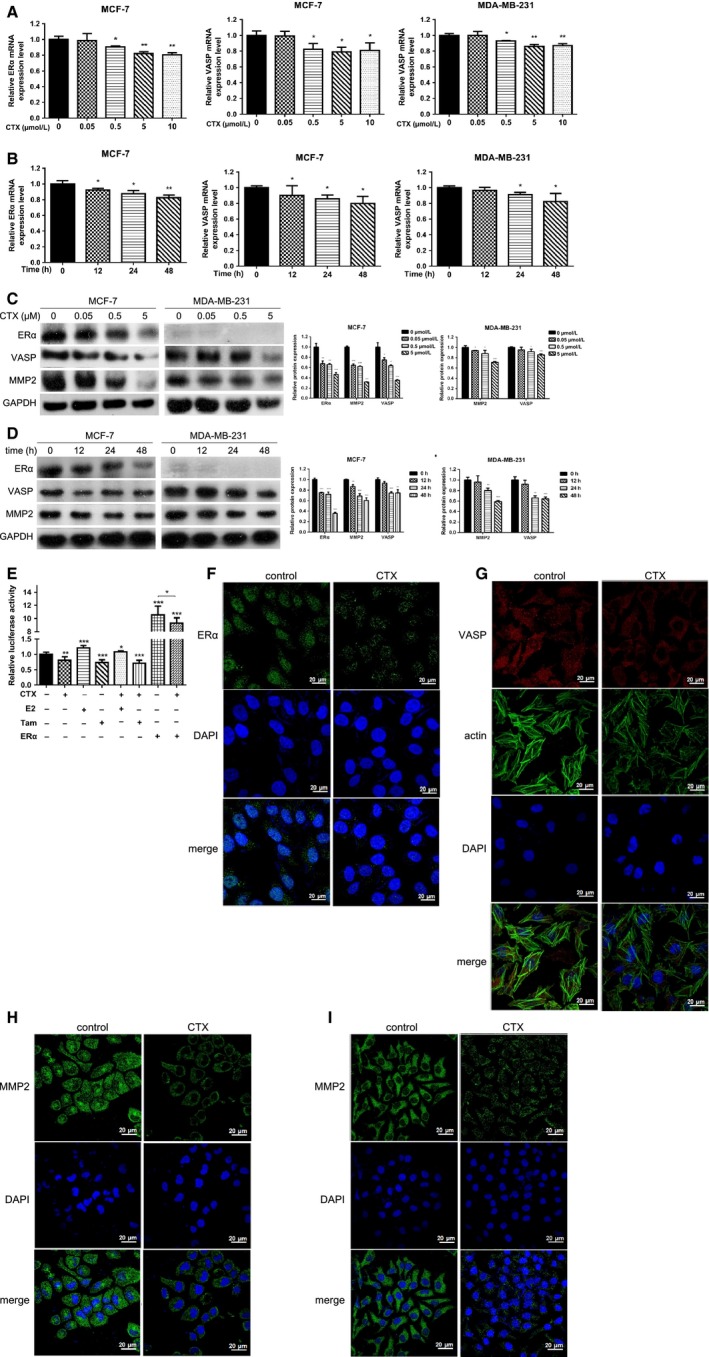
CTX can significantly inhibit the expression levels of ERα and VASP in breast cancer cells. (A) MCF‐7 and MDA‐MB‐231 cells were treated with 0, 0.05, 0.5, 5, and 10 μmol/L CTX for 24 hours and harvested for detecting the mRNA expression levels of ERα and VASP by RT‐qPCR. (B) MCF‐7 and MDA‐MB‐231 cells were treated with 5 μmol/L CTX for 0, 12, 24, and 48 hours, respectively, and harvested for detecting the mRNA expression levels of ERα and VASP by RT‐qPCR. (C) MCF‐7 and MDA‐MB‐231 cells were treated with 0, 0.05, 0.5, and 5 μmol/L CTX for 24 hours, and harvested for detecting the protein expression levels of ERα and VASP by western blotting. (D) MCF‐7 and MDA‐MB‐231 cells were treated with 5 μmol/L CTX for 0, 12, 24, and 48 hours, respectively, and harvested for detecting the protein expression levels of ERα and VASP by western blotting. (E) The effect of CTX, E2, Tam, and ERα overexpression on the activity of ERα promoter reporter gene was detected by a luciferase reporter gene assay in MCF‐7 cells. MCF‐7 (F‐H) and MDA‐MB‐231 (I) cells were treated with 5 μmol/L CTX for 24 hours. The effect of CTX treatment on the expression and distribution of ERα, VASP, MMP2, and actin was detected by immunofluorescence. *P < 0.05, **P < 0.01, ***P < 0.001. CTX, chlorotoxin; ERα, estrogen receptor α; RT‐qPCR, quantitative reverse transcription polymerase chain reaction

To observe the effects of different concentrations of CTX on the expression of ERα and VASP mRNA in MCF‐7 and MDA‐MB‐231 breast cancer cells, cells were treated with 0, 0.05, 0.5, 5, and 10 μmol/L CTX for 24 hours, and harvested for detecting the mRNA expression levels of ERα and VASP by RT‐qPCR. The results showed that in MCF‐7 cells, compared with the control group, when treated with CTX at 0.05, 0.5, 5, and 10 μmol/L, the mRNA expression level of ERα was decreased at 1.3%. 9.5%, 18.2%, and 19.5%, respectively (*P* < 0.05). In MCF‐7 and MDA‐MB‐231 cells, the inhibition rates of VASP mRNA expression levels were 0.8%, 17.6%, 21.1%, and 19.2%, and 0%, 6.8%, 13.9%, and 13.1%, respectively (*P* < 0.05) (Figure 3A). In addition, cells treated with 5 μmol/L CTX for 0, 12, 24, and 48 hours, respectively, the results showed that in MCF‐7 cells, compared with the control group, the inhibition rates of ERα mRNA expression were 7.7%, 12.5%, and 17.3%, respectively (*P* < 0.05). In MCF‐7 and MDA‐MB‐231 cells, compared with the control group, the inhibition rates of VASP mRNA expression were 1.0%, 13.8%, and 20.2%, and 3.6%, 8.9%, and 17.5%, respectively (*P* < 0.05) (Figure 3B).

In addition, western blotting results showed that in MCF‐7 cells, the inhibitory rates of CTX for ERα, VASP, and MMP2 proteins at 0.05, 0.5, and 5 μmol/L were 32.0%, 33.7%, and 53.0%, and 25.2%, 36.3%, and 64.9%, and 35.4%, 37.6%, and 68.1%, respectively (*P* < 0.05). In MDA‐MB‐231 cells, the inhibition rates of VASP and MMP2 protein expression were 5.0%, 8.4%, and 14.8%, and 6.2%, 12.1%, and 29.3%, respectively, compared with the control group (*P* < 0.05) (Figure 3C). In addition, cells treated with 5 μmol/L CTX for 0, 12, 24, and 48 hours, respectively. The results showed that in MCF‐7 cells, compared with the control group, the inhibition rates of ERα, VASP, and MMP2 protein expression were 24.6%, 27.7%, and 64.5%, and 6.9%, 25.4%, and 25.2%, and 13.1%, 31.2%, and 39.4%, respectively (*P* < 0.05). In MDA‐MB‐231 cells, the inhibition rates of VASP and MMP2 protein expression were 8.3%, 34.1%, and 35.7% and 4.2%, 20.1%, and 40.8%, respectively (*P* < 0.05), compared with the control group (Figure 3D). Furthermore, another ER‐positive breast cancer cells T47D was also treated with 0, 0.05, 0.5, and 5 μmol/L CTX for 48 hours. The expression of ERα, MMP2, and VASP were detected by western blotting (Figure S1C). These results indicate that CTX can inhibit the mRNA and protein expression of ERα, MMP2, and VASP in a time‐ and concentration‐dependent manner in MCF‐7, MDA‐MB‐231, and T47D cells.

Using the luciferase reporter gene assay, we found that CTX was able to significantly inhibit the activity of ERα promoter reporter gene and reverse the activated effect of E2 and ERα overexpression on the ERα promoter reporter gene in MCF‐7 cells (*P* < 0.05) (Figure 3E).

To observe the effect of CTX treatment on the expression and distribution of ERα, VASP, MMP2, and actin in breast cancer cells, MCF‐7 and MDA‐MB‐231 cells were treated with 5 μmol/L CTX for 24 hours. Protein expression, distribution, and subcellular localization were detected by immunofluorescence. The results showed that compared with the control group, the expression of total ERα protein in the CTX group was decreased, the expression level of ERα in nuclear was decreased, the expression level of VASP and actin protein was decreased, the morphology of actin was irregular and loose, and the expression level of MMP2 protein was also decreased (Figure 3F‐I).

### 3.4 CTX can directly interact with ERα and affect the protein secondary structure of ERα‐LBD

**Figure 4:**
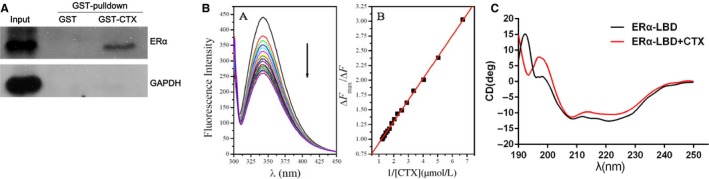
CTX can directly interact with ERα and affect the protein secondary structure of ERα‐LBD. The binding of CTX and ERα was detected by a pull‐down assay (A) and internal fluorescence emission spectroscopy assay (B). The effect of CTX on the secondary structure of ERα‐LBD was detected by a circular dichroism assay (C). CTX, chlorotoxin; ERα, estrogen receptor α

To further explore the relationship between CTX and ERα, we tested the binding of CTX and ERα by pull‐down assay. The results show that CTX could directly interact with ERα (Figure 4A). Furthermore, we examined the binding of CTX to ERα‐LBD by internal fluorescence emission spectroscopy. The results show that CTX in the range of 0.27‐0.82 μmol/L can quench the ERα fluorescence emission signal and tend to be saturated. The calculated binding constant of CTX and ERα‐LBD was 3 × 10^7^ L/M, which was slightly lower than of 3 × 10^9^ L/M between E2 and ERα (Figure 4B). The results indicated that CTX and ERα had strong binding effect.

To investigate the effect of CTX on the secondary structure of ERα‐LBD, we detected by circular dichroism assay, and found that ERα‐LBD showed a low peak at 222 and 208 nm, and a high peak near 190 nm, suggesting the structure of α‐helix exists in the ERα‐LBD domain, which is also consistent with previous studies. After treated with CTX protein, the lowest peak at 222 nm and the highest peak at 190 nm disappeared (Figure 4C), suggesting that the number of α‐helix in the secondary structure of ERα‐LBD protein was decreased.

### 3.5 VASP was a target gene of ERα signaling pathway

**Figure 5:**
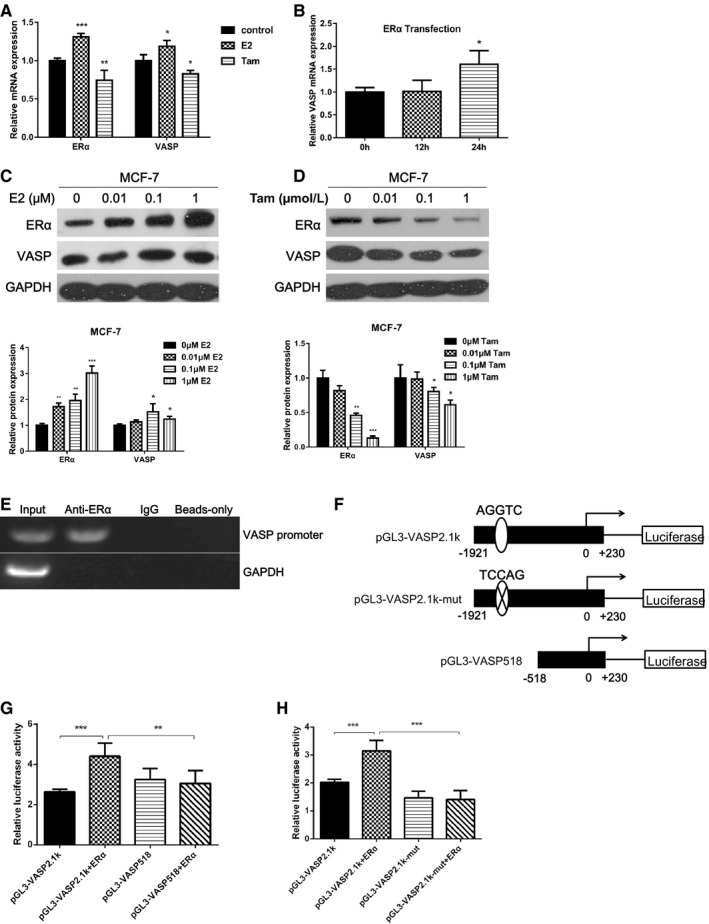
VASP was a target gene of ERα signaling pathway. (A) E2 or Tam was used to activate or inhibit the ERα signaling pathway, respectively. In addition, the mRNA expression of ERα and VASP was detected by RT‐qPCR. (B) After transfection of ERα overexpression plasmid, VASP mRNA expression was detected by RT‐qPCR. (C) MCF‐7 cells were treated with 0, 0.01, 0.1, and 1 μmol/L E2 for 24 hours. The protein expression levels of ERα and VASP were detected by western blotting. (D) MCF‐7 cells were treated with 0, 0.01, 0.1, and 1 μmol/L Tam for 24 hours, the protein expression levels of ERα and VASP were detected by western blotting. (E) ERα can bind to the promoter of VASP, which was detected by ChIP assay. (F) The wild‐type and mutated ERα binding site, and the truncated of VASP promoter reporter plasmids were constructed. (G) The effect of ERα overexpression on the activity of wild‐type or truncated VASP promoter reporter, which was detected by luciferase reporter gene assay. (H) The effect of ERα overexpression on the activity of wild‐type and mutated ERα binding site of VASP promoter reporter, which was detected by luciferase reporter gene assay. *P < 0.05, **P < 0.01, ***P < 0.001. ChIP, chromatin immunoprecipitation; ERα, estrogen receptor α; RT‐qPCR, quantitative reverse transcription polymerase chain reaction

To observe the effect of ERα signaling pathway on VASP expression, we used E2 or Tam to activate or inhibit the ERα signaling pathway, respectively. The results showed that after E2 treatment, the mRNA expression of ERα and VASP increased by 31.1% and 18.8%, respectively, while the mRNA expression of ERα and VASP in the Tam treatment group decreased by 25.7% and 17.4%, respectively (*P* < 0.05) (Figure 5A). In addition, after transfection of ERα overexpression plasmid, VASP mRNA expression was increased by 60.7% (*P* < 0.05) (Figure 5B). Then, we treated the cells with 0, 0.01, 0.1, and 1 μmol/L E2 for 24 hours. The protein expression levels of ERα and VASP was increased by 71.7%, 95.5%, and 201.7%, and 13.2%, 52.0%, and 23.4%, respectively (*P* < 0.05) (Figure 5C). After treatment with 0, 0.01, 0.1, and 1 μmol/L Tam for 24 hours, the protein expression levels of ERα and VASP were decreased by 18.4%, 54.0%, and 86.8% and 1.3%, 19.6%, and 38.8%, respectively (*P* < 0.05) (Figure 5D). Furthermore, the effect of 1 μmol/L Tam, 5 μmol/L CTX, and 1 μmol/L Tam combined with 5 μmol/L CTX on the proliferation, migration, and invasion of MCF‐7 cells was detected by CCK‐8 and transwell assays (Figure S2A,B). The results showed that 5 μmol/L CTX had the same inhibitory effect with 1 μmol/L Tam (*P* > 0.05), while 5 μmol/L CTX combined with 1 μmol/L Tam could produce a stronger inhibitory effect (*P* < 0.05).

Through bioinformatics prediction and ChIP assay, it was found that ERα can bind to the promoter of VASP (Figure 5E). Next, we designed a site‐directed mutagenesis primer for AGGTC targeting the core binding sequence to mutate AGGTC to TCCAG, as well as different lengths of VASP promoter reporter plasmids (Figure 5F). The luciferase reporter gene results showed that ERα overexpression significantly increased the pGL3‐VASP2.1 k reporter gene activity (*P* < 0.05), but had no significant effect on pGL3‐VASP518 (*P* > 0.05) (Figure 5G). In addition, the reporter gene activity of the pGL3‐VASP2.1k‐mut group was significantly decreased relative to the pGL3‐VASP2.1k group (*P* < 0.05) (Figure 5H). The above results indicate that the ERα binding site is located between the 1921 and 518 bp regions upstream of the transcription start site of VASP, wherein the AGGTC sequence is the core binding site of ERα in the VASP promoter region.

### 3.6 CTX can target ER‐positive breast tumors in vivo

**Figure 6:**
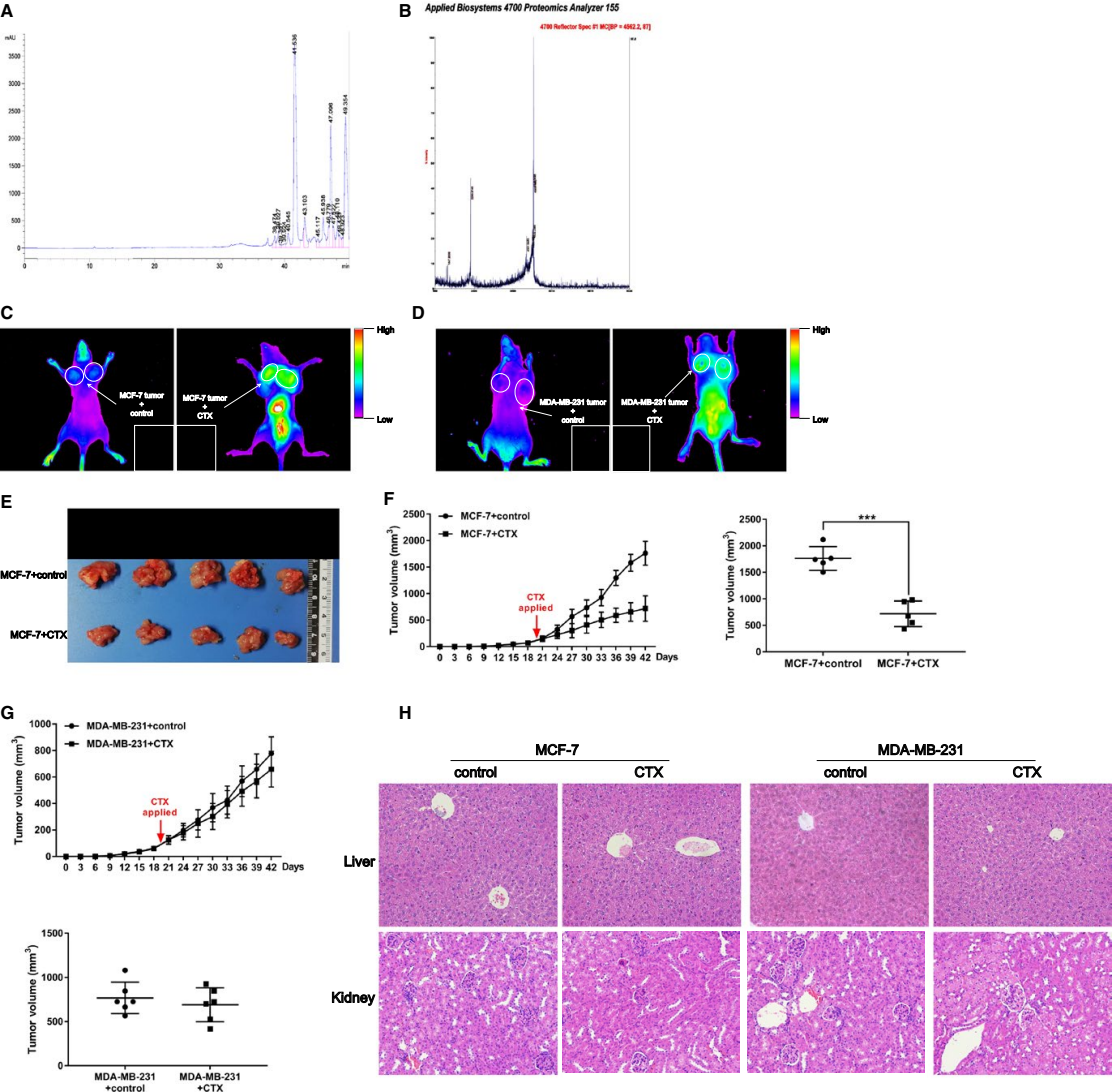
CTX can target ER‐positive breast tumors in vivo. (A) The CTX: Cy5.5 recombination protein was purified by RP‐HPLC. (B) The molecular weight of the purified CTX: Cy5.5 recombination protein was determined by MALDI‐TOF‐MS method. MCF‐7 (C) and MDA‐MB‐231 (D) breast cancer cells were transplanted into nude mice, respectively, and the distribution of CTX in mice was observed by small animal in vivo imaging technique. (The arrow and circle indicated the location of tumors in vivo, and the square indicated the tumors ex vivo.) The effect of CTX on MCF‐7 tumor (E) and the growth curve and weight (F) of tumors were present. (G) The effect of CTX on the growth curve and weight of MDA‐MB‐231 tumors were present. (H) The liver and kidney tissues of nude mice were collected and detected by H&E staining. ***P < 0.001. CTX, chlorotoxin; ER, estrogen receptor; RP‐HPLC, reversed‐phase high‐performance liquid chromatography; H&E, hematoxylin and eosin

To observe the effect of CTX on the growth of orthotopic xenografts in breast cancer, we constructed and successfully prepared a CTX: cy5.5 fusion protein (Figure 6A,B). Cy5.5 is a near‐infrared dye that is often used to label peptides, proteins, and oligonucleotide amino groups for subsequent small animal in vivo imaging experiments. In this study, we injected MCF‐7 and MDA‐MB‐231 breast cancer cells into nude mice, respectively, and when the transplanted tumor grew to about 150 mm3, CTX: Cy5.5 was injected through the tail vein. Then, the distribution of CTX in mice was observed by a small‐animal in vivo imaging technique (Figure 6C,D). It was found that CTX has a more significant enrichment effect in MCF‐7 tumors than MDA‐MB‐231 breast tumors, and CTX can significantly inhibit the growth rate and weight of MCF‐7 tumors (*P* < 0.05) (Figure 6E,F). However, there was no significant difference in the volume and weight of MDA‐MB‐231 tumors between the control group and the CTX treatment group (*P* > 0.05) (Figure 6G). In order to evaluate the effect of CTX on liver and kidney in nude mice, we collected liver and kidney tissues of nude mice in each group. After H&E staining, no obvious abnormalities were found in the liver and kidney tissues between the control group and CTX treatment group (Figure 6H).

## 4 DISCUSSION

Breast cancer is a serious threat to women's health, and the incidence rate is increasing year by year, and it shows a trend of rejuvenation. Despite the continuous improvement in the diagnosis and treatment of breast cancer in recent years, the mortality rate of breast cancer patients is still not effectively controlled.^1^ Because of the rapid proliferation of breast cancer, the ability to metastasize distant organs, and the resistance to more and more therapeutic drugs, its treatment problems have attracted much attention. In recent years, small molecule peptide anti‐tumor drugs have attracted more and more attention.^25^ In the preliminary work of this study, we found that the small molecule peptide CTX can significantly inhibit the proliferation, migration and invasion ability of breast cancer cells, suggesting its potential as a therapeutic drug for breast cancer, but its mechanism of action remains unclear.

In recent years, studies on ER have focused on two aspects: regulation of cell proliferation and cell invasion. The ER signaling pathway plays an important role in the tumorigenesis and development. When estrogen activates the ER signaling pathway, it stimulates the expression of various genes involved in cell proliferation, causing malignant tumors such as breast cancer.^26^ Other studies have shown that ER can regulate the invasion and metastasis of tumor cells.^27^ Therefore, targeting ER signaling pathway is one of the important strategies for breast cancer treatment. In this study, we found that the small molecule peptide CTX can significantly inhibit the expression of ERα, suggesting that CTX can play a role in the development of breast cancer by inhibiting ER signaling pathway. Further studies have found that CTX can directly interact with ERα and affect the number of α‐helix in the LBD domain of ERα, and thus change its secondary structure. The LBD domain is a ligand binding region of ERα and is closely related to the transcriptional regulation function of ERα.^28^ ERα‐LBD consists of 12 α‐helix structure (H1–H12) and one β‐sheet structure, which is very susceptible to other external factors such as ligands and drugs.^29^ The conformational changes in this region directly affect the biological function of ERα. Therefore, our results suggest that CTX can not only inhibit the expression level of ERα, but also inhibit the function of ER signaling pathway by changing the secondary structure of ERα protein.

ERα is activated to form an ERα homologous or heterodimer, which enters into the nucleus and binds to a specific estrogen response element to regulate transcription of downstream target genes.^30^ In this study, we found that the promoter region of VASP contains an ERα binding site by bioinformatics prediction combined with molecular level validation. The activity of ERα signaling pathway is positively correlated with the expression level of VASP, suggesting that VASP is a new target gene of ERα signaling pathway. VASP is an actin‐related skeletal protein that plays an important role in regulating tumor cell migration and invasion. VASP promotes the assembly and elongation of F‐actin by promoting the insertion of actin monomer into the assembly end of F‐actin, thereby regulating cell movement and migration process, promoting invasion and metastasis of multiple tumors, including breast cancer.^31^ Our previous series of studies have demonstrated the key role of VASP in regulating the proliferation, migration and invasion of various tumor cells such as breast cancer.^32^, ^33^, ^34^ In the present study, we found that CTX can inhibit ER signaling pathway and VASP expression, and also inhibit colocalization of VASP with actin, affecting F‐actin assembly and aggregation, causing cytoskeletal dysfunction, thereby inhibiting the proliferation and migration of breast cancer cells MCF‐7 and T47D. Furthermore, in addition to being regulated by the ERα signaling pathway, the expression of VASP was also closely related to various signaling pathways or transcription factors.^34^, ^35^, ^36^ Therefore, although ER was negative in MDA‐MB‐231 cells, CTX could still inhibit VASP expression, which may be through other mechanisms.

In addition, many studies have used CTX as a target platform for the treatment of tumors, which can play a role in clinical tumor therapy. CTX can target MMP2 protein on the surface of glioma and inhibit MMP2 activity.^21^ Based on CTX‐131I coupling, the ^131^I‐TM‐601 radioactive probe has been approved by the FDA as the first anti‐human glioma drug that has entered the phase I/II clinical trial stage.^37^, ^38^ The complex of fluorescent dyes such as Cy5.5 and CTX for tumor targeting allows visualization of tumor location during surgery.^39^ In vivo glioma can be detected by MRI using a complex of CTX and superparamagnetic iron oxide nanoparticles.^40^ CTX and platinum complexes can target a variety of tumor cells.^24^ In mouse breast cancer cell 4T1, based on the CTX‐MMP2 coupling mechanism, CTX can enhance the targeted killing effect of doxorubicin on 4T1 cells.^41^ In the present study, we found that compared to ER‐negative MDA‐MB‐231 tumors, CTX has a stronger targeting effect on ER overexpressing MCF‐7 tumors. Compared with MDA‐MB‐231 cells, the expression of MMP2 in MCF‐7 cells is low. Therefore, the targeting effect of CTX on MCF‐7 tumors may be related to the interaction between CTX and ERα. These results suggest that based on the targeting effect of CTX on ERα, CTX can directly exert its antibreast cancer effect, and can also be used as a carrier in combination with other antitumor drugs to treat breast cancer more accurately.

**Figure 7:**
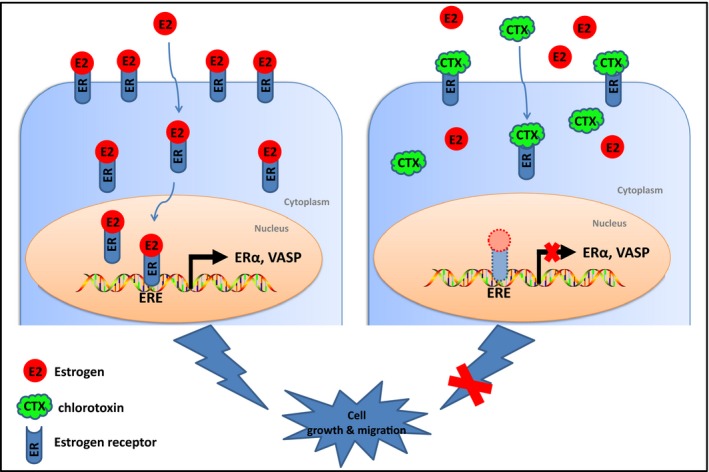
Working model for the regulation of proliferation and migration of breast cancer cells by CTX. CTX can directly interact with ERα to inhibit the expression of ERα, which inhibits the ERα/VASP signaling pathway, leading to suppression of cell growth and migration in breast cancer. CTX, chlorotoxin; ER, estrogen receptor

In summary, we found that CTX can inhibit the progression of breast cancer. CTX can directly interact with ERα to inhibit the ERα/VASP signaling pathway by inhibiting the expression level of ERα and altering its protein secondary structure (Figure 7). In in vivo study, we found that CTX is more inclined to target ER‐positive breast tumors. Based on this, our study reveals a new mechanism of CTX anti‐ER‐positive breast cancer, which also provides an important reference for the study of CTX anti‐ER‐related tumors.

## CONFLICT OF INTEREST

None declared.
