# Supplementary figures and images for: Chlorotoxin targets ERα/VASP signaling pathway to combat breast cancer
Source: Cancer Med. 2019 Feb 25;8(4):1679–93. doi: 10.1002/cam4.2019 (PMC6488122; doi:10.1002/cam4.2019)

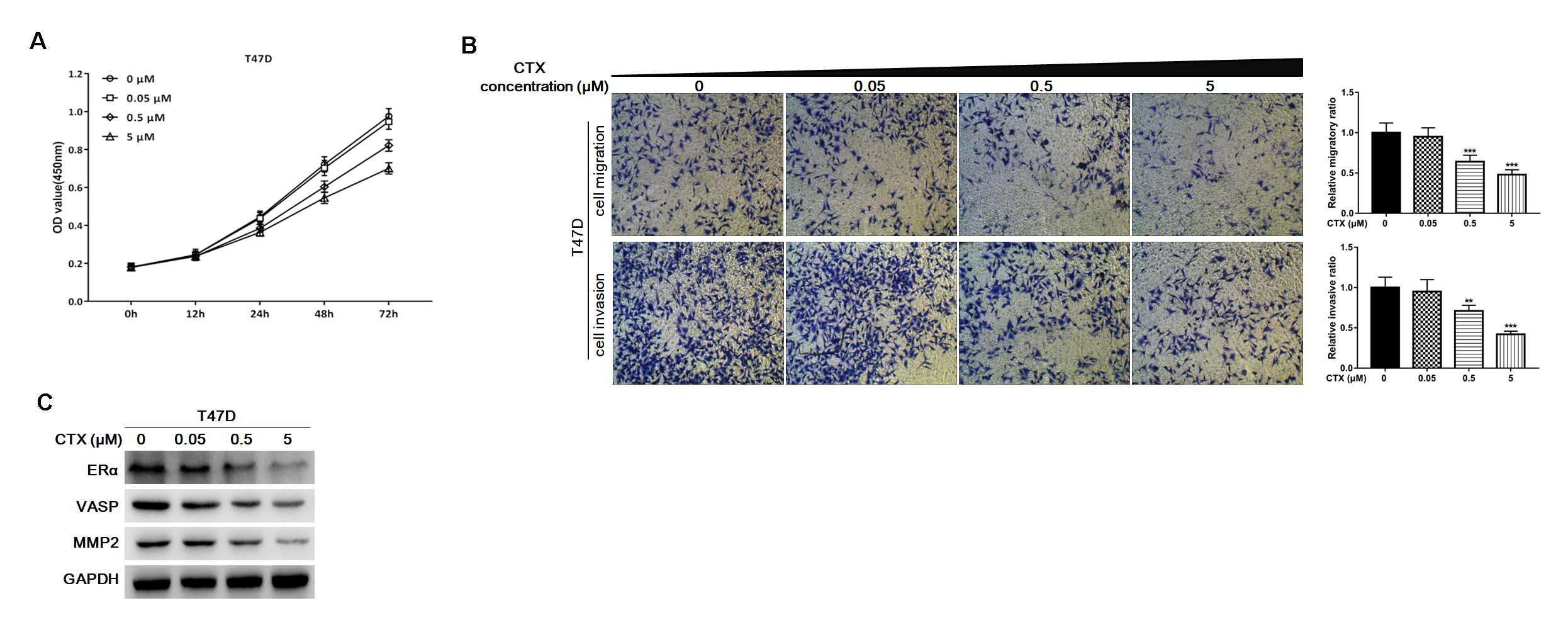

Supplement: Supplementary file 1 [file CAM4-8-1679-s001.tif]

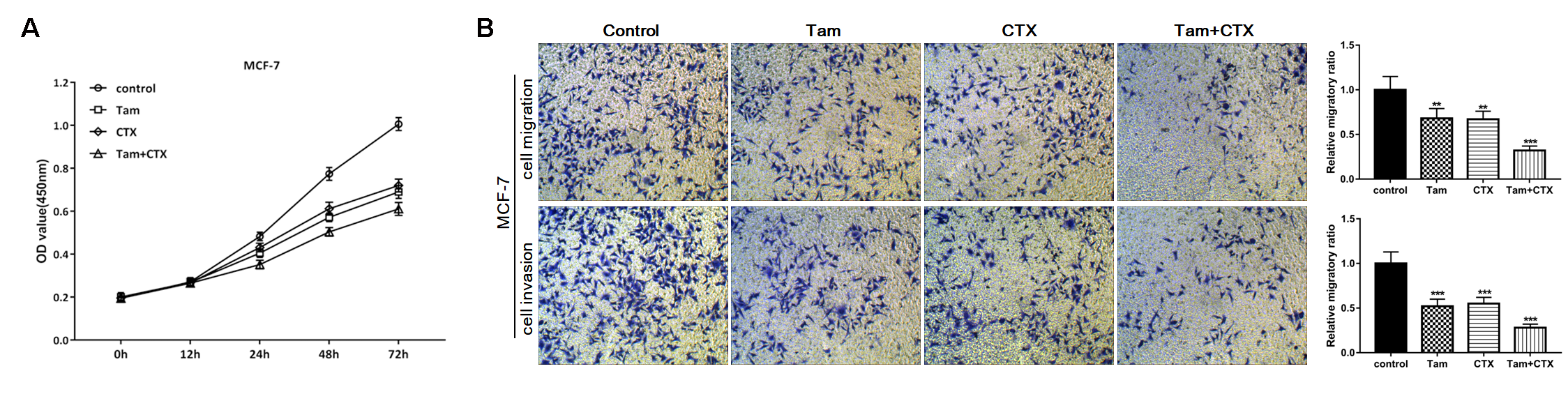

Supplement: Supplementary file 2 [file CAM4-8-1679-s002.tif]
